# Feeling the burn in the era of COVID-19: cross-cultural adaptation and validation of the Arabic version of the Copenhagen Burnout Inventory among community pharmacists

**DOI:** 10.1186/s40545-022-00419-x

**Published:** 2022-03-17

**Authors:** Dalal Youssef, Linda Abou-Abbas, Janet Youssef

**Affiliations:** 1 Bordeaux Research Center for Population Health, Institut de santé publique, d’épidémiologie et de développement (ISPED), Bordeaux University, Bordeaux, France; 2 Clinical trial Program, Ministry of Public Health, Beirut, Lebanon; 3 Neuroscience Research Center, Faculty of Medical Sciences, Lebanese University, Beirut, Lebanon; 4 Epidemiological Surveillance Program, Ministry of Public Health, Beirut, Lebanon; 5 Al Zahraa Hospital University Medical Center, Beirut, Lebanon

**Keywords:** Validation, Psychometric, Arabic version, Copenhagen Burnout Inventory, Community pharmacists

## Abstract

**Background:**

Community pharmacists (CPs) are one of the frontline healthcare workers (HCWs) working diligently to provide much-needed services during the COVID-19 pandemic. Burnout was one of the detrimental outcomes of the pandemic on the mental health of Lebanese CPs. To assess the extent of this syndrome among Lebanese CPs, a psychometrically reliable and valid tool is needed.

**Objectives:**

This study aimed to validate the Arabic version of the Copenhagen Burnout Inventory (CBI-A) for use in the assessment of burnout among CPs.

**Methods:**

A web-based cross-sectional study was conducted among Lebanese CPs over February 2021. Data were collected using an anonymous Arabic self-administered questionnaire that includes information on socio-demographic characteristics, work-related variables, in addition to the measurements: the CBI which includes personal, work-related, and patient-related dimensions of burnout, and the hospital anxiety and depression scale. Data were analyzed using SPSS and Amos software. Exploratory factor analysis and confirmatory factor analysis were performed to explore the factorial structure and to measure model fit. Cronbach’s alpha was used to assess internal consistency. The criterion validity of the CBI was assessed. Multivariable linear regression analyses were used to explore the association between different aspects of burnout and mental health outcomes such as depression and anxiety.

**Results:**

The CBI-A showed high internal consistency with Cronbach’s alphas varied from 0.774 to 0.902 and a low floor and ceiling effect (1–9%). As for the CBI-A construct validity, the exploratory factor analysis showed three factors with good factor loadings and explained 72.17% of the variance. The confirmatory analysis supported the three-factorial structure of the CBI that presented a good overall fit based on the goodness-of-fit indices. Ad hoc modifications to the model were introduced based on the modification indices to achieve a satisfactory fit by allowing one covariate error between one pair of items within the personal burnout domain. All of the 19 items were kept in the construct since they showed a good factorial weight. The CBI-A is associated with burnout-related factors in expected directions, including extensive working hours, sleeping hours, and job satisfaction, indicating, therefore, the criterion validity of the tool. CBI subscales were also found positively associated with mental health outcomes such as depression and anxiety demonstrating, in turn, a predictive validity.

**Conclusion:**

This study provides evidence for the validity and reliability of the Arabic version of CBI as an adequate tool for assessing burnout among CPs. Such an instrument could be useful for assessing such syndrome among other healthcare workers.

**Supplementary Information:**

The online version contains supplementary material available at 10.1186/s40545-022-00419-x.

## Background

Burnout syndrome is one of the occupational problems gradually faced in recent years that captured the attention of modern societies [1]. It was defined as an occupational phenomenon associated with long-lasting workplace stress that was not successfully managed. The latter can affect the health status of the concerned individual or his interaction with health services [2]. One of the well-known pieces of evidence on burnout is that it can occur in high-risk professions [3]. These demanding jobs and work environments usually required plugging more emotional and mental efforts and consuming more time for providing services [4]. However, these additional requirements were faced by a profession offering scarce opportunities and limited job security.

Similar to other demanding jobs, burnout syndrome is, unfortunately, a comorbidity affecting all disciplines of the healthcare and community-pharmacy personnel in all practice settings are no exception [5–7]. It is often associated with time constraints and performance metrics [8]. Throughout the years, the role of CPs has evolved considerably to cover new autonomous services such as immunizations and medication therapy management. However, this pivot from product-based care to service-based added additional layers of complexity to the profession including managing the delicate balance of incorporating clinical services into traditional dispensing environments [9, 10].

With the emergence of COVID-19, the professional role of the CPs has progressed considerably which required an adaptation to the model of care [11]. As the most accessible healthcare professionals, CPs have been recognized during the pandemic as essential front-liners. They were able to maintain the continuity of healthcare services, and to undertake additional responsibilities that alleviate pressure on other health services [12]. In addition, CPs turned out to be an information hub about COVID-19 through dispelling rumors and misinformation flood regarding medicines, sharing accurate information, and advising patients about healthy behaviors [13]. Of note, the COVID-19 pandemic comes amidst a long-standing appetite for further professional role development. It has been an opportunity for CPs to integrate a bridge between medical care and wider community services which represented an area of promise for the future [14]. However, these inflicted duties on the shoulder of community-pharmacy personnel in the wake of COVID-19 have created ideal conditions to leave this valuable human resource at increased risk of burnout in the aftermath [15]. Of note, the significance of burnout lies in its negative physical and mental health outcomes such as cardiovascular diseases and obesity as well as anxiety and depression [16]. It can also affect CP’s job performance, decrease productivity and quality of services, increase absenteeism, and can lead to job dissatisfaction, low organizational commitment, intention to leave the job, and staff loss [17]. Results of previous studies among pharmacists exhibited links between workload, time pressure, role conflict, role ambiguity, job satisfaction, type of pharmacy, and burnout level in addition to individual characteristics such as age, sex, marital status [6–10].

Although several studies were performed for assessing this syndrome among healthcare workers, there is a dearth of comparable data on the prevalence of burnout and its associated factors. This could be due to different definitions of the syndrome and the heterogeneity of assessment methods. The Maslach Burnout Inventory (MBI), which is only commercially available, has been used, so far, in the majority of the studies assessing burnout [18–20]. However, this concept was reviewed and a new instrument called Copenhagen Burnout Inventory (CBI) was developed by Kristensen et al. This instrument, which was translated into eight languages, allows assessing burnout in different settings with three sub-dimensions: Personal burnout (PB), work-related burnout (WB), and client-related burnout (CB) and provides better accuracy in the approach to the work environment [21, 22]. The CBI adds a new aspect related to burnout related to personal life (PB) which allows the comparison of burnout among individuals regardless of occupational status [21, 23].

The Lebanese context was ideal for instigating burnout among CPs who played a major role in medication management during the pandemic [24]. During a period where in-person healthcare consultations are reserved, the public turned to CPs as they remain the most accessible face-to-face primary healthcare provider. In addition, Lebanese people, as a proactive step, to prepare for a conceivable infection by COVID-19, rushed to the community pharmacies to purchase supportive drugs and food supplements. Similar to other countries, Lebanon’s supply chain of goods, including medical supplies, was also deeply impacted by the pandemic [25]. However, while many other countries have managed to cushion the impact of COVID-19, Lebanon has thus far been incapable of doing so as it struggles with a steep loss of the value of the Lebanese currency combined with the inflation of the prices of the medicines [26]. Furthermore, several local and potentially manageable factors such as the smuggling of subsidized medications outside of the country, the stockpiling of medications by patients and local warehouses in anticipation of future pricing hikes, and the delayed processing time for subsidies by “Banque du Liban” (BDL) [27, 28] have also contributed to this crisis. All the above-mentioned conditions combined with the required precautionary measures against COVID-19 at pharmacy premises created typical conditions to leave burned-out pharmacy personnel in the aftermath. However, burnout in the landscape of the CPs population is rarely explored. This could be due to the absence of recognized free-of-charge validated measurement tools. Therefore, adapting and validating CBI among Lebanese pharmacists is of great interest since it will support the use of CBI, a free-of-charge burnout inventory for assessing burnout among healthcare workers.

This study aimed to assess the psychometric properties of the Arabic version of the Copenhagen Burnout Inventory (CBI) for use in the assessment of burnout among Lebanese CPs and to explore the association between different aspects of burnout and mental health outcomes such as depression and anxiety.

## Methods

### Study design and population

Using a non-probability convenience sampling technique, a web-based cross-sectional study was carried out among Lebanese community pharmacists during February 2021. Contacts details of the potential participants were obtained from the list of registered CPs provided by the Lebanese order of pharmacists (OPL). Participants were electronically invited to participate through an online questionnaire. A link to the study was shared with CPs through emails and WhatsApp. Two weeks after the initial contact and link sharing, a reminder was sent to the CPs previously recruited.

All CPs of either gender (male, female) or profile (owner, manager, or staff pharmacist) working in the Lebanese community pharmacies and having access to the internet and willing to contribute to the study were eligible for participation. However, retired CPs, clinical pharmacists, those who were out of the country at the time of the survey, trainees and pharmacy students or other pharmacies staff (dietician…), as well as those not practicing actually, were excluded from the study. In addition, CPs suffering from psychiatric or psychological illnesses were also excluded.

All methods were performed following the relevant guidelines and regulations. No remuneration was given to the CPs for their involvement in the study which was voluntary. All information were collected anonymously and handled confidentially. None of the survey’s queries questioned for information that could harm the respondent in any way. Informed consent was obtained in an electronic format.

### Sample size calculation

The CBI scale consisted of 19 items. As suggested by Comrey and Lee [29], the minimal sample size required to perform a confirmatory factor analysis was 190 based on ten participants for each item. However, to increase the power of the study and to reduce the sampling error and increase the study power, a rough estimation was made by multiplying the calculated sample size by 2.03 times, leading to a final sample size of 387 participants.

### Instrumentation

#### Validation of the Arabic version of CBI among pharmacists

Our primary objective was to validate the CBI among community pharmacists. The Arabic/English version was adapted to include three main domains with 19 items that cover personal burnout along with work-related and client-related burnout. The validation of A-CBI psychometric proprieties comprised the following working steps.

##### Forward–backward translation

The original 19-item version of CBI [21] was meticulously translated from English to the Arabic language by two masked certified bilingual translators who were selected independently from the English Literature and Arabic Literature Departments in one of the Lebanese Universities. Inconsistencies found between the two translators were discussed. Then, the initial translated version was back-translated by 2 independent translators who are native speakers of the English language [30]. A committee of experts was composed to identify and verify linguistic, problematic items and discrepancies in terms of wording, and the ambiguity of the CBI to ensure authenticity and reach consensus over ambiguous terminologies [31]. A consensus was reached on keeping all the CBI items leading to the pre-final version of the translated CBI which is piloted on a small sample of 35 HCWs. Based on the feedback of respondents participating in the pilot testing, minor revisions including the change of confusing wording to the lay language were made to address potentially misleading items and ambiguous terminologies.

### Reliability

*Internal consistency reliability* The reliability of the CBI was evaluated using internal consistency which looks at the consistency of the score of individual items on an instrument, with subscales. The internal consistency reliability is estimated using Cronbach’s alpha (*α* ≥ 0.70 was considered satisfactory) [32]. Of note, group variability, number of items, the difficulty level of the tool, and sample size could impact Cronbach’s alpha value.

*Test–retest reliability* For the test–retest reliability which measures the correlation between scores from one administration of an instrument to another, 22 CPs were asked to fill out the questionnaire for the second time after almost 3 weeks, this duration between the first test and the retest aimed to avoid artificial reliability resulting from memory bias. In terms of sample size required, when alpha and power are fixed at 0.05 and lower than 80%, respectively, a minimum sample size of 22 is considered sufficient. Since test–retest reliability is commonly conducted during the initial pilot study, a small sample size is usually required. Test–retest reliability was evaluated using Pearson correlation ((Pearson’s *r*) where its value ≥ 0.70 was considered satisfactory for ruling on the correlation between the retest and the initial study.

### Validity

#### Face and content validity

To assess face validity, two separate Likert scales were used to evaluate clarity and comprehension. The former was evaluated with a 5-point scale that varied from 1 to 5 (for example, 1 for totally incomprehensible to 5 for easy to understand). The face validity index was the average index value of the above indexes. The results were then converted in values between 0 (totally unclear or incomprehensive) to 1 (clear or understandable). A face validity index above 80% was considered satisfactory in the present study (Additional file 1: Appendix S2) [33].

As for content validity, it was assessed to ensure the necessity of each item in the collected sample using qualitative and quantitative methods. The panel of experts which consisted of the psychologist (one), epidemiologists (two), community pharmacists (two), and an occupational health specialist (one), was asked to review the potential scale items and validate the appropriateness of these items as indicators of the construct (burnout). Assessment of the item aspects, in terms of the level of clarity, relevance, applicability, comprehensiveness, and ease of understanding is performed using the method proposed by Lawshe [34] using 5 points Likert scale varying from 1 to 5 (for example 1: not clear to 5: very clear). For qualitative evaluation, a few items were substituted with other simpler texts.

For quantitative evaluation, we estimated both Content Validity Index (CVI) and content validity ratio (CVR). To obtain the content validity index for relevancy and clarity of each item, the Item Validity Index (I-CVIs) was estimated as follows: the number of those judging the item as relevant or clear (rating 3 or 4) was divided by the number of panelists. In terms of relevance, the Content Validity Index at the scale level (S-CVI) is determined by estimating [The sum of relevant proportional rating/(number of experts)].

To calculate an item CVR, the following formula is used: CVR = (*Ne* − *N*/2)/(*N*/2) [34]

In this ratio, *Ne* is the number of panelists (content experts) who indicated that this item is “essential” and *N* is the total number of panelists. The mean CVR of all items computes an overall CVR. It is recommended for a scale with good content validity to be composed of I-CVIs of 0.78 or higher and S-CVI of 0.8 and 0.9 [35].

### Floor and ceiling effects

Scale items were also assessed for determining the questionnaire sensitivity by calculating the bottom (Floor) effects and roof (Ceiling) effects. The ceiling and flooring effects were calculated by the percentage of the lowest or the highest possible score achieved by respondents. The floor and ceiling effects of more than 15% were considered to be significant [36].

### Construct and factorial validity

Factorial validity was assessed by the definition and evaluation of the domain structure of the A-CBI questionnaire using models of exploratory factor analysis.

The two tests of Kaiser–Meyer–Olkin (KMO) and Bartlett were performed before factor analysis. KMO measure for sampling adequacy and a value greater than 0.6 (Mediocre value) depict the appropriateness of conducting factor analysis [37, 38]. Bartlett’s test of sphericity was used to test the identity of correlation matrices and significant values affirm a satisfactory factor analysis [38].

We split the original sample into two random samples containing approximately half of the participants, one for exploratory analysis and the second for confirmatory analysis. To determine whether the original CBI, including 19 items, was reliable and valid for the Lebanese CPs, and to identify CBI dimensions, the first sample (*N* = 190), was subjected to principal component analysis, and the items were exposed to factor analysis with Varimax rotation. Domains enrolled in each model were selected based on Kaiser’s criterion (eigenvalues > 1), graphical analysis of scree plot, and the total variance explained (at least greater than 50%), it was decided on the number of factors to be included in the model. Then, we performed a parallel analysis (PA) to determine the number of components or factors to retain from factor analysis [39]. To evaluate the internal consistency of the CBI, Cronbach’s alpha reliability coefficients were calculated.

Furthermore, a confirmatory factor analyses (CFA) were performed using IBM AMOS 24.0. The following fit indices and the respective cut-off for the goodness of fit have been reported to assess the construct validity of the questionnaire. The structural models were considered as a good fit to the data when: (1) having a good absolute fit measured using the root mean square error of approximation (RMSEA < 0.08), the standardized root mean square residual (SRMR < 0.08) and Goodness-of-Fit Index (GFI) with a level of acceptance more than 0.9 [40]. (2) having a good incremental fit which was calculated using Comparative Fit Index (CFI), Tucker–Lewis Index (TLI), and Normed Fit Index (NFI), all of them  were expected to be more than 0.9 [41, 42] (3) Lastly, having a good parsimonious fit was examined by Chi-squared value/degree of freedom (Chisq/*df*) which should be less than 5 [43]. Factor loading values of 0.3 and more were considered a significant relationship between items and factors. In case of a poor fit, re-specification was guided by considerations of the theoretical underpinnings of the CBI, and inspection of modification indices, and standardized residuals to assure substantive justification and to improve the goodness of fit of the models [44]. Covariances were permitted to be freely estimated. Items that cross-loaded on two or more factors will be eliminated in the modified model [44].

### Criterion validity

Criterion validity of the CBI items was assessed by testing correlations between the CBI and other factors associated with burnout; extensive working hours, sleep disturbance, and job satisfaction. A negative correlation was hypothesized between desirable job satisfaction and high burnout level, and a positive correlation was hypothesized between excessive workload, sleep disturbance, and high burnout level.

### Predictive validity

The correlation between CBI and relevant mental health outcomes that could result from burnout was such as anxiety and depression.

### Questionnaire development

Using google form, an online anonymous self-administrated questionnaire was developed in the Arabic and the English languages. The completion of the questionnaire took around 9–12 min to complete. It included mainly closed-ended questions and consisted of four sections: (a) socio-demographic characteristics; (b) CP lifestyle; (c) occupational factors, and (d) the measurements. The first section collected socio-demographic data of the participants, including gender, age, marital status, profile, education level, residency, and health status. It also included questions about the history of medical illnesses, the health status of people living with the participant, and the presence of an elderly or dependent child at home. The second section covered the topic of CP’s lifestyle where participants were asked about their sleep pattern, their physical activity, and their tobacco and alcohol consumption. The third section enclosed occupational factors and the exposure to COVID-19. CPs were queried to answer on whether they have worked in the frontline, dealing with COVID-19 patients, working extensive hours, and their job satisfaction as well. In terms of exposure to COVID-19, CPs were asked if they have been tested for COVID-9, been diagnosed as COVID-19 case, had a family member relative or colleague infected by COVID-19. Each of these variables was answered on a yes or no basis. The fourth section consisted of two scales to objectively assess anxiety and depression, and burnout among the CPs.

1. The Hospital Anxiety and Depression Scale (HADS)

The HADS, a 14-item questionnaire, is a frequently used self-rating scale, designed for anxiety and depressive disorders. It consisted of two subscales assessing anxiety (7 items) and depression (7 items), which are rated on a 4-point Likert-type (from 0 to 3) [45]. The scores in each subscale are computed by summing the corresponding items, with maximum scores of 21 for each subscale. A score of 0–7 is considered as normal, 8–10 as a borderline case, and 11–21 as a case of mood disorder or pathology (anxiety or depression). In this study, we used the Arabic version of the HADS which has been validated in several Arab countries such as Saudi Arabia [46], Kuwait [47], and the United Arab Emirates [48] in both emergency care primary-care settings and. Overall, it has demonstrated satisfactory psychometric properties in different groups and the general population. The reliability of HADS in the current study was 0.814.

2. The Arabic version of Copenhagen Burnout scale A-CBI

The 19-item CBI version was used in the current study to evaluate personal-related (6 items), work-related (7 items), and client-related (6 items) burnout [21]. The questions of CBI are mixed with questions on other topics to avoid stereotyped response patterns. CPs were asked to rate how often they felt exhausted. Ratings were given based on a five-point Likert scale. Each item was scored from 0 to 100 (0 = never, 25 = seldom, 0 = sometimes, 75 = often, 100 = always). Of note, some questions were answered using another five-point Likert scale (to a very high degree, to a high degree, somewhat, to a low degree, to a very low degree). Mean items score was calculated per scale.

### Statistical analysis

The generated data on an excel spreadsheet were transferred to the statistical software IBM SPSS® software (Statistical Package for Social Sciences) version 24.0 for analysis. Given that the response of all questions was mandatory, there was no missing data to substitute. For descriptive analysis, frequency and percentage were used for categorical variables, the mean and standard deviation for quantitative variables. The normality distribution of CBI scale items was confirmed by calculation of skewness and kurtosis values which are lower than 1 [49]. Floor and ceiling effects were described as a percent. Reliability and validity were assessed using the aforementioned appropriate tests. The Student’s *T*-test was used to compare the means between two groups, whereas one-way analysis of variance ANOVA to compare between three groups or more, after checking for homogeneity of variances. A Pearson’s correlation was applied to link used scores with burnout subscales. All variables that showed a *p*-value < 0.2 in the bivariate analysis were included in the multivariable analysis as an independent variable. Four regressions using the stepwise method were conducted to identify the correlates of each of the CBI scales, after checking the absence of multicollinearity. *p* < 0.05 was considered statistically significant.

## Results

### Baseline information of the participants

Out of the 387 CPs participating in this study, more than half of them were female (53.7%), married (60.5%), and living in urban areas (65.9%). The mean age for the study sample was 45 years (*SD* = 11.0) and ranged from 25 to 71 years. In terms of educational level and professional experience, 55.8% of surveyed CPs hold a BS degree in pharmaceutical sciences, and 43.2% of them had a large practical experience (> 10 years). The majority of CPs worked more than 40 h per week (59.9%), in pharmacies located in the Mount-Lebanon governorate. More than three-quarters of them had a good health status. In terms of living conditions, more than 50% of them had a child, elderly, or a family member with comorbidities living with them at home (59.4%). Around one-quarter of respondents reported a previous history of COVID-19 infection. A detailed description of the baseline characteristics of the surveyed community pharmacists is presented in Additional file 1: Appendix S1.

### Description of the scale

Descriptive statistics for the CBI items and subscales are reported in Table 1. The descriptive statistics for the CBI items showed no substantial violation of the condition of normality required for CFA since skewness and kurtosis were within acceptable levels based on a cut-off of >|1| and the sample size larger than 300. Average scores and standard deviations were described for each item per scale and the scale as a whole measure to the Arabic version. No missing values were reported. All the dimensions had low values of bottom and ceiling effects (< 15%).

**Table 1:** Summary statistics of CBI

	CBI items	Mean	*SD*	Floor %	Ceiling %	Skewness	Kurtosis
PB1	How often do you feel tired?	66.41	16.56	2%	4%	− 0.37	− 0.56
PB2	How often you are physically exhausted?	66.41	16.56	3%	5%	− 0.19	− 0.32
PB3	How often you are emotionally exhausted?	66.41	16.56	6%	4%	0.27	0.98
PB4	How often do you think: “I can’t take it anymore”?	69.06	12.48	1%	3%	0.64	0.28
PB5	How often do you feel worn out?	67.38	16.88	4%	8%	0.42	0.91
PB6	How often do you feel weak and susceptible to illness?	67.38	16.88	3%	6%	− 0.75	− 0.66
WB1	Is your work emotionally exhausting?	64.08	13.42	5%	4%	− 0.12	− 0.33
WB2	Do you feel burnt out because of your work?	68.67	11.18	4%	7%	0.67	0.38
WB3	Does your work frustrate you?	69.12	11.91	2%	4%	0.40	0.28
WB4	Do you feel worn out at the end of the working day?	69.77	11.53	6%	5%	0.34	0.19
WB5	Are you exhausted in the morning at the thought of another day at work?	66.15	12.24	3%	3%	− 0.35	− 0.71
WB6	Do you feel that every working hour is tiring for you?	69.51	11.26	6%	6%	− 0.92	− 0.23
WB7	Do you have enough energy for family and friends during leisure time?^R^	66.60	12.09	9%	8%	0.26	0.63
CB1	Do you find it hard to work with clients?	68.27	21.39	7%	4%	− 0.35	− 0.56
CB2	Do you find it frustrating to work with clients?	62.82	22.39	6%	9%	− 0.82	− 0.73
CB3	Does it drain your energy to work with clients?	67.67	23.53	4%	5%	0.27	0.58
CB4	Do you feel that you give more than you get back when you work with clients?	68.82	24.29	8%	7%	0.43	0.88
CB5	Are you tired of working with clients?	72.03	24.98	4%	6%	0.52	0.61
CB6	Do you sometimes wonder how long you will be able to continue working with clients?	69.30	21.23	5%	8%	0.31	0.78

*SD* standard deviation, *%* percentage, *R* reverse coding

### Face validity

With regard to face validity, the universal validity index was 88.53%, while clarity was 85.96%, and comprehension was 91.11%. Further analyses per subscale and question are shown in Additional file 1: Appendix S1. The aforementioned values indicate sufficient face validity.

### Content validity

For quantitative measurment of content validity index and ratio to the scales holistically; S-CVI [0.87; ranged (0.72–0.96)] and CVR [0.82; ranged (0.71–0.98)] showed satisfactory results.

### Construct validity

#### Factor analysis

KMO test result (KMO = 0.883) was satisfactorily indicating good sampling and Bartlett’s test was highly significant (*p* < 0.001). As a result of factor analysis, using Varimax rotation, items converged over a solution of three factors that had eigenvalues over 1.0 and the examination of the scree plot suggested that the three-factor solution was the most interpretable one. Of note, the total variance explained was 72.17%. Parallel analysis (PA) informed us also that three factors surpassed the PA criterion, which explained also 81% of the total variance. Both analyses yielded the same results in terms of the higher factor coefficient for each of the items selected. According to the Varimax rotated matrix, the loadings of the 19 items on each of these four factors are presented in Table 2. The items for each factor were similar to those in the original scale. As a result, the first factor with 7 items was called ‘‘work-related burnout”, accounting for the variance of 33.34%, and had an eigenvalue of 6.34. The second factor, called “client-related burnout” included 6 items. It was responsible for 23.54% of the total variance and had an eigenvalue of 3.044. These items describe patient-related items that instigate CPs burnout. Factor 3, called “personal burnout” consisted of 6 items, which was responsible for 15.28% of the total variance and had an eigenvalue of 1.841 (Table 2).

**Table 2:** Three-factor solution of CBI items, eigenvalues, and Cronbach’s alpha coefficients, and variance of the CBI subscales among community pharmacists

	CBI scale items	CBI components		
		Work burnout (*N* = 7 items)	Client burnout (*N* = 6 items)	Personal burnout (*N* = 6 items)
WB1	Is your work emotionally exhausting?	0.881		
WB3	Does your work frustrate you?	0.845		
WB4	Do you feel worn out at the end of the working day?	0.813		
WB6	Do you feel that every working hour is tiring for you?	0.803		
WB5	Are you exhausted in the morning at the thought of another day at work?	0.781		
WB2	Do you feel burnt out because of your work?	0.729		
WB7	Do you have enough energy for family and friends during leisure time?	0.707		
CB2	Do you find it frustrating to work with clients?		0.801	
CB3	Does it drain your energy to work with clients?		0.792	
CB1	Do you find it hard to work with clients?		0.732	
CB6	Do you sometimes wonder how long you will be able to continue working with clients?		0.673	
CB4	Do you feel that you give more than you get back when you work with clients?		0.652	
CB5	Are you tired of working with clients?		0.618	
PB6	How often do you feel weak and susceptible to illness?			0.782
PB3	How often are you emotionally exhausted?			0.761
PB5	How often do you feel worn out?			0.727
PB1	How often do you feel tired?			0.714
PB2	How often are you physically exhausted?			0.701
PB4	How often do you think: “I can’t take it anymore”?			0.691
*E*	Eigenvalue	6.335	3.044	1.841
*α*	Cronbach alpha (overall CBI = 0.861)	0.902	0.774	0.842
*V*	Variance	33.342	23.546	15.283

Factor loadings below 0.30 were omitted for the sake of clarity, 72.17% of the variance was explained, extraction method: principal component analysis. Rotation method: Varimax with Kaiser normalization

#### Scales reliabilities and intercorrelation between CBI subscales

Reliability and summary statistics for CBI subscales for the Arabic version are illustrated in Table 3. In this sample of CPs, overall burnout had a mean of 65.34 (*SD* = 17.39) while the value for personal burnout, work-related burnout, and client-related burnout scales were 67.17 (*SD* = 16.82), 67.02 (*SD* = 14.15), and 69.38 (*SD* = 20.78), respectively. All the used scales showed good reliability; CBI (*α* = 0.868); PB (*α* = 0.842); work-related burnout (*α* = 0.902), and client-related burnout (*α* = 0.774). CPs revealed a similar level of personal and work-related burnout aspects. However, client-related burnout ranked first among other dimensions of burnout reported by CPs.

**Table 3:** Summary statistics for the Arabic version of Copenhagen Burnout Inventory subscales

#		Number of items	Item mean	*SD*	Min	Max	*α*	Skewness	Kurtosis
D1	Personal burnout	6	67.17	16.82	63.89	71.93	0.842	− 0.375	− 0.566
D2	Work-related burnout	7	67.02	14.15	63.89	69.93	0.902	− 0.192	− 0.323
D3	Patient burnout	6	69.38	20.78	52.70	79.09	0.774	0.267	0.938
	Overall CBI score	19	65.34	17.39	59.17	70.09	0.868	0.640	0.928

*SD* standard deviation, *Min* minimum, *Max* maximum, *α* Cronbach alpha

#### Confirmatory factor analysis

**Fig. 1:**
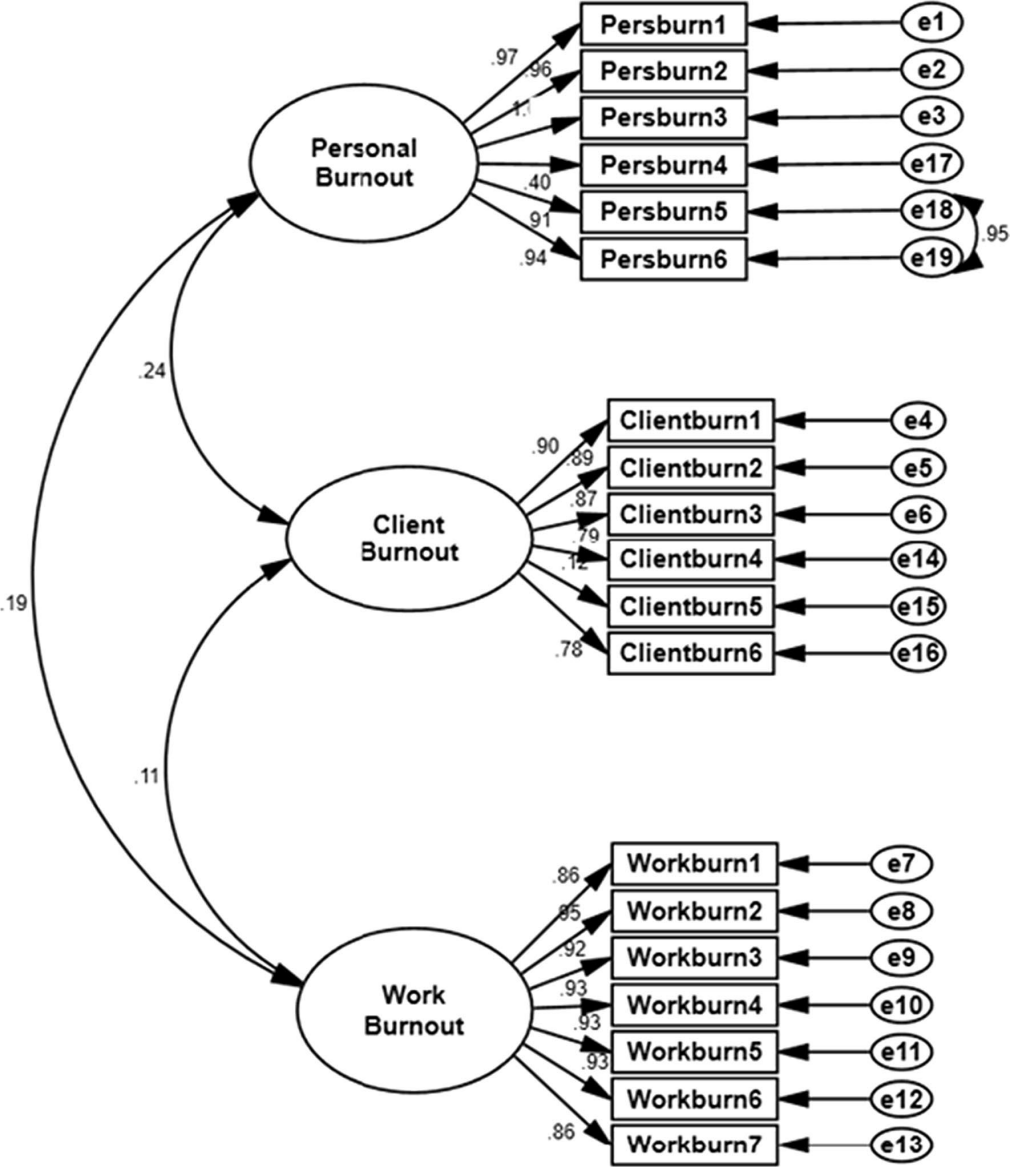
Confirmatory analysis of the CBI factorial structure

Three models were tested by confirmatory analysis. The first model was the one-factor model was found to have an inadmissible solution on account of the presence of factor loading greater than 1.0 and a negative variance between items. The second model was the default model corresponding to the result of the exploratory analysis that does not fit our data. Although model 2 was found to have an admissible solution with a slightly better fit than model 1, an unacceptable RMSEA level informed inspection of the model for localized areas of poor fit. Large values of modification indices, expected parameter change values, and standardized residuals revealed the possible omission of noticeable indicator error correlations between some items. One model specification was performed to achieve a better fit. We covaried the items as follows: item 5 “How often do you feel worn out?” and item 6 in the personal burnout dimension: “How often do you feel weak and susceptible to illness?”. This was deemed to make satisfactory substantive sense (Fig. 1). This resulted in a better fitting than the default model. Thus, model 3 was retained as the final solution. A summary of goodness-of-fit indices for different measurement models is displayed in Fig. 1. The model fit measures of the data analysis of the model 3 were as follows (*χ*^2^/*df* = 4.46; NFI = 0.927, CFI = 0.917, GFI = 0.918, RSMR = 0.042, RMSEA = 0.041 < 0.08), therefore suggesting a reasonable model fit (Table 4).

**Table 4:** Summary of goodness-of-fit indices for different measurement models

Variable	*χ*^2^statistic (*df*)	Cmin/*df*	RMSEA	RMSEA CI	SRMR	GFI	CFI	NFI	TLI	Comments
Model 1: one-factor model	2013 (195)	10.32	0.111	(0.191–0.218)	0.089	0.681	0.683	0.678	0.687	Inadmissible solution
Model 2: three-factor model	843.34 (121)	7.147	0.072	(0.083–0.112)	0.055	0.901	0.902	0.908	0.907	Admissible solution with poor fit
Model 3: three-factor model with covariates	522.37(117)	4.46	0.041	(0.063–0.088)	0.043	0.918	0.917	0.927	0.911	Admissible solution: retained model With a satisfactory fit

Model 1: 1 factor and 19 items, Model 2: 19 items and the three original factorial structures, Model 3: 19 items, original three-factorial structure, and the covariation between PB 5 and PB 6 items

#### Intercorrelation between CBI and its dimensions

As shown in Table 5, there were statistically significant correlations ranging from *r* = 0.212 (*p* < 0.01) to *r* = 0.377 (*p* < 0.01) between the subscales of CBI. As expected, there were moderate-to-high positive correlations between the CBI scale and its 3 dimensions. The highest correlation was between the CBI scale and client burnout (*r* = 0.719, *p* < 0.01) followed by CBI and work-related burnout (*r* = 0.520, *p* < 0.01). The CB and the WB subscales were moderately correlated.

**Table 5:** Correlation matrix for the Arabic version of Copenhagen Burnout Inventory subscales and HADS

	Personal burnout	Work-related burnout	Client-related burnout	Overall CBI scale	HADS anxiety	HADS depression
Personal burnout (PB)	1.000	0.212**	0.297**	0.4 43**	0.597**	0.464**
Work-related burnout (WB)		1.000	0.377**	0.520**	0.488**	0.414**
Client burnout (CB)			1.000	0.719**	0.401**	0.319**
Overall CBI				1.000	0.552**	0.401**
HADS anxiety					1.000	0.378**
HADS depression						1.000

**p < 0.01 significance level

#### Correlation between CBI subscales, HADS depression and HADS anxiety

HADS anxiety scale was significantly correlated to each of the 3 dimensions of CBI. The highest correlation was observed between PB and anxiety (*r* = 0.597, *p* < 0.01). Similarly, HADS depression was also significantly correlated with each aspect of burnout as follows: depression-PB: *r* = 0.44, *p* < 0.01; depression-WB, *r* = 0.414, *p* < 0.01; depression-CB: *r* = 0.319, *p* < 0.01 (Table 5).

#### Association between baseline characteristics and CBI subscales

Female CPs, those who are young (less than 40 years), and working as staff pharmacists had a significantly higher level of burnout than their counterparts. Similarly, CPs having limited professional experience, extensive working hours, insufficient sleeping hours, and those who expressed their dissatisfaction towards their works expressed also a higher level of burnout. These factors were found also significantly associated with a high level of PB, WB, and CB except the age which was not significantly associated with the work-burnout dimension. The largest effect size was observed in age, marital status, and presence of a dependent child at home (Table 6).

**Table 6:** Association between baseline characteristics and CBI subscales (*N* = 387)

	*N* (%), *N* = 387	Overall CBI			Personal burnout			Work-related burnout			Client-related burnout		
		Mean (*SD*)	*p* value	Eta squared	Mean (*SD*)	*p* value	Eta squared	Mean (*SD*)	*p* value	Eta squared	Mean (*SD*)	*p* value	Eta squared
Gender			0.036	0.027		0.048	0.013		0.312	0.001		0.022	0.08
Male	179 (46.25%)	65.85 (7.02)			65.78 (14.55)			66.98 (10.02)			66.71 (15.47)		
Female	208 (53.74%)	66.94 (6.01)			68.5.6 (15.54)			67.06 (12.22)			72.05 (17.48)		
Age (years)			0.018	0.071		0.022	0.09		0.021	0.072		0.039	0.05
Less than 40 years	254 (65.6%)	67.35 (6.02)			67.37 (15.66)			65.14 (10.32)			67.44 (17.21)		
≥ 40 years	133 (34.4%)	63.33 (7.09)			62.99 (15.84)			68.91 (11.82)			71.31 (16.81)		
Profile			0.003	0.12		0.041	0.013		0.003	0.092		0.018	0.04
Staff pharmacist	135 (34.9%)	68.23 (10.98)			70.23 (14.89)			69.32 (10.21)			72.32 (14.98)		
Owner/manager	252 (65.1%)	62.45 (9.72)			64.11 (10.78)			64.72 (11.38)			66.44 (13.21)		
Years of experience			0.048	0.015		0.213	0.001		0.001	0.06		0.031	0.02
Less than 10 years	220 (56.9%)	64.38 (10.23)			68.15 (14.32)			70.15 (14.23)			70.32 (16.28)		
≥ 10 years	167 (43.2%)	66.40 (10.18)			66.19 (16.16)			63.89 (13.28)			60.5 (16.58)		
Extensive working hours			0.014	0.041		0.049	0.014		0.018	0.03		0.048	0.009
No	155 (40.1%)	63.25 (9.81)			65.45 (12.37)			63.91 (12.99)			67.78 (15.91)		
Yes	232 (59.9%)	67.43 (10.93)			68.89 (11.76)			70.13 (12.23)			70.98 (15.58)		
Sleeping hours			0.009	0.06		0.037	0.018		0.003	0.12			
Less than 6 h	167 (43.2%)	70.32 (9.38)			69.32 (10.18)			69.98 (11.23)			70.13 (12.06)		
More than 6 h	220 (56.8%)	60.36 (10.21)			65.02 (11.87)			64.0 (12.32)			68.63 (10.98)		
Job satisfaction			0.049	0.032		0.018	0.032		0.041	0.07		0.048	0.032
Satisfied	167 (43.2%)	63.55 (11.03)			64.13 (10.85)			63.21 (9.81)			67.53 (12.22)		
Not satisfied	220 (56.8%)	67.13 (10.23)			70.21 (9.32)			70.83 (10.78)			71.23 (13.82)		

*N* frequency, *%* percentage, *SD* standard deviation

#### Association between burnout and other mental health outcomes

In terms of burnout dimensions, PB [*β* = 0.096, CI 95% (0.076–0.192)], WB [*β* = 0.246, CI 95% (0.102–0.379)] and CB [*β* = 0.083, CI 95% (0.098–0.138)] were significantly associated with higher depression. Similarly, PB [*β* = 0.141, CI 95% (0.036–0.197)], WB [*β* = 0.198, CI 95% (0.089–0.221)] and CB [*β* = 0.041, CI 95% (0.066–0.134)] were significantly associated with higher level of anxiety (Table 7).

**Table 7:** Linear regression: burnout and mental health outcomes (depression and anxiety)

Dependent variable: depression				
1st linear regression: depression as a dependent variable				
	Unstandardized *β*	Standardized *β*	*p* value	CI 95%
Burnout subscales				
Personal burnout	0.18	0.096	0.011	(0.076–0.192)
Work-related burnout	0.34	0.246	0.024	(0.102–0.379)
Client-related burnout	0.11	0.083	0.041	(0.098–0.138)

Dependent variable: anxiety				
2nd linear regression: anxiety as a dependent variable				
	Unstandardized *β*	Standardized *β*	*p* value	CI 95%
Burnout subscales				
Personal burnout	0.185	0.141	0.031	(0.036–0.197)
Work-related burnout	0.198	0.163	0.018	(0.089–0.221)
Client-related burnout	0.085	0.041	0.048	(0.066–0.134)

*CI* confidence interval

## Discussion

Burnout syndrome should be a focus of concern in healthcare especially in the context of COVID-19 because of its impact on the physical and psychological well-being of frontlines healthcare workers. From this perspective, measurement and diagnostic tools that are adequately calibrated to the healthcare workers' population must be available. Since the wide usage of the CBI and its aspects in the assessment of main aspects of burnout was mainly based on face validity with limited empirical evidence on its structural validity, it seems necessary to investigate in depth the latent structure of this instrument. Therefore, the current study is the first nationwide study that aimed to examine the validity (latent structure) and the reliability of the Arabic version of the CBI, a free-of-charge burnout inventory, for assessing burnout syndrome in community pharmacies in Lebanon.

The main findings of our study were that the CBI has good psychometric properties and could be used for the assessment of burnout among CPs. The CBI-A showed high internal consistency and the Cronbach’s alphas varied from 0.774 to 0.902. Low floor and ceiling effects were also found. As for CBI-A construct validity, the exploratory factor analysis showed three factors with good factor loadings and explained 72.17% of the variance. The confirmatory analysis supported the three-factorial structure of the CBI which presented a good overall fit revealed by the goodness-of-fit indices. Based on the modification indices, The adapted three-factor model, allowed one covariate error between one pair of items within the PB domain (PB5: How often do you feel worn out? and PB6: How often do you feel weak and susceptible to illness?). All of the 19 items were kept in the construct since they showed a good factorial weight. The CBI-A is associated with burnout-related factors in expected directions, including extensive working hours, sleeping hours, and job satisfaction, indicating criterion validity. CBI subscales were found also positively associated with mental health outcomes such as depression and anxiety indicating a predictive validity.

As for the tool’s reliability, our findings showed a high internal consistency of the CBI-A and the Cronbach’s alphas varied from 0.774 to 0.902. Similar results were reported by other studies conducted in different countries and settings such as New Zealand [50], Taiwan [51], China [52], Spain [53], Portugal, and Brazil [54]. This uniformity and steadiness of findings, in terms of the CBI’s internal consistency, across countries, contexts, and languages, demonstrated its internal structural stability of this public domain measurements [55] and shed light on the opportunity to broadly expand its use and its applicability in further settings than the ones originally proposed by Kristensen et al.

Similar to the findings of other studies conducted in Iran [56] and Serbia [57], low floor and ceiling effects (1–9%) were also found in our study. As for CBI-A face validity which was the first step in analyzing the psychometric properties of the instrument, all items of the CBI-A were easily understood by CPs, as shown by the face validity index values, which were more than 80%, hence indicating satisfactory face validity. Despite the disparity in the targeted populations in other studies assessing burnout using CBI, our results were comparable to their findings in terms of validity [21, 58]

In terms of content validity, the CBI-A was also considered very satisfactory after achieved minor modifications based on experts’ suggestions. Of note, some participants mentioned that some items seemed to be repetitive such as ‘How often do you feel tired out?’ and ‘How often do you feel physically exhausted?’. This could be due to the challenges encountered during the process of translation when trying to find appropriate and equivalent terms for these expressions. Some words such as ‘tired’, ‘physically exhausted’, ‘psychologically exhausted’, and ‘worn-out’ all sounded almost the same in the Arabic language. Similar challenges were previously reported by other studies conducted in China and Serbia [51, 57]. This finding underlines the need to take into account any possible cultural difference that could be related to the exhibition of burnout in future studies. However, the CBI-A is reflected to be a proper response procedure that evidenced its validity based on Cook et al.’s current concepts in validity and reliability for psychometric instruments despite the presence of such an effect [59].

As for CBI-A’s construct validity, the EFA showed three factors with good factor loadings and explained more than 70% of the variance. The factors completely corresponded to the subscales in the original instrument where all of the items in the WB scale loaded in the same factor (factor 1), as were all of the items regarding CB (factor 2) and those related to PB (factor 3). The confirmatory analysis (CFA) supported the three-factorial structure of the CBI-A and presented a good overall fit. All goodness-of-fit indices also supported the model construct validity. As all the 19 items showed a good factorial weight, hence all the 19 items were kept. Of note, ad hoc modifications to the model were introduced to achieve a satisfactory fit by allowing for only one error covariances within PB items (PB5: How often do you feel worn out? and PB6: How often do you feel weak and susceptible to illness?) based on modifications indices. Such covariance of two contiguous items could be ensued from similar wording and could lead to a similarity in participants’ understanding of a specific situation. Of note, several studies exploring the latent structure of the CBI using either EFA or CFA ensued in inconclusive results. Our results were consistent with the findings of some studies that provided empirical evidence supporting the initial three-factor solution to fit the data and the differentiation of the three distinct domains of the CBI as well as the adequacy of such factorial structure [50, 52, 58, 60, 61]. While some researchers such as Javanshir et al. who removed some of the CBI items [56] to achieve a satisfactory fit of data and to support the three-factorial structure of the CBI, other scientists were driven in similar studies to introduce ad hoc modifications [52, 58, 60–62]. The mentioned re-specifications included allowing several error covariances both within pairs of items as well as between dimensions [52, 58]. However, inflation of some relevant fit indices could result from such modifications, which, in turn, could lead to questioning the factorial validity of the tool. This was not the case in our study as only one covariate between adjoining items of the same domain.

In terms of intercorrelation between CBI and its three dimensions, as expected a moderate-to-high positive correlations were found in our study. The highest correlation was revealed between the CBI-A and its CB subscale. This was foreseeable among Lebanese CPs given the increase in demand for medicines from clients combined with the limited supply chain, the COVID-19 threat, and the required precautionary measures at pharmacy premises. All these factors created typical conditions to leave burned-out pharmacy personnel in the aftermath.

As for CBI-A subscales correlations, there were statistically moderate positive correlations between WB and CB. A possible explanation could be that many CPs spend the majority of their time at the workplace and, therefore, in continuous contact with clients. This can slightly disturb participants’ perception of separating client and work-related burnout. Such correlations between these factors were rarely reported in previous studies. However, the weak correlation between PB and WB found in our study could be explained that disengaged CPs may feel exhausted from their daily work but not worn out when they leave work which is not the case of workaholic CPs who may feel exhausted in daily life but not weary of their work.

In terms of criterion validity, our findings showed a positive association in the expected direction between extensive working hours, sleep disturbance, and CBI subscales. A statistically significant negative association was also found between job satisfaction and CBI items, in the hypothesized direction confirmed the criterion validity for the CBI. In addition, increasing age was found in our study as a protective factor against burnout. Using the adapted CBI-A to assess burnout in a population of Lebanese CPs, the risk of high burnout in all its aspects appeared to decrease with increasing age, with CPs younger than 40 years having the highest risk of high burnout level in all its dimension. Similarly, CPs with limited work experience suffered from a higher level of burnout. This trend is consistent with the findings of a study conducted among pharmacists in the United States [63]. Such a result could be explained by the fact that older CPs learned from their life experience and previous encounters with stressful events how to anticipate, cope and prepare for potentially tough situations. Therefore, they are better than younger CPs in engaging in their work, applying positive adaptation and emotion management skills. The gender difference was also revealed in terms of PB and CB. Female pharmacists appeared at a high risk of exhibiting a higher level of burnout than males. Similar results were found in a study conducted among pharmacy practice faculty in the US [64].

In terms of mental outcomes, mental health outcomes such as depression and anxiety were found associated with CBI subscales indicating a predictive validity of CBI. People who suffered from burnout look would act as if they were depressed [65]. The occurrence of anxiety and depression can negatively impact the way the individual copes with daily stressors, which may be related to the use of ineffective strategies to manage stress. Similarly, emotional exhaustion was found by Ding et al. to be positively associated with anxiety symptoms [66].

Lastly, it is worth mentioning that the average scores on the PB, WB, and CB scales found in our study were higher than those demonstrated previously in studies examining burnout [67–69]. The high burnout level in all CBI subscales could be related to the Lebanese context where the country has been recently crippled by several crises including the economic downfall, the COVID-19 pandemic, and the Beirut blast which is ranked the most powerful non-nuclear explosion of the twenty-first century.

In summary, the CBI-A could be used to measure burnout across three dimensions—personal burnout, work-related burnout, and client-related burnout. Our validation process confirmed the three-factor structure for burnout to function adequately in pharmacists and derived a parsimonious instrument.

## Limitations of the study

There are some limitations to be acknowledged in this study. Selection bias was conceivable as we used a convenient sampling technique. The latter could limit the generalizability of the findings. To minimize the possible influence of this bias, upcoming studies should randomly select participants. However, participants have nearly the same characteristics of the Lebanese CPs population in terms of sex, age, and geographical distribution, therefore, therefore the best scenario in terms of representativeness of CPs despite the non-probability sampling technique used for data collection. In addition, the cross-sectional design of our study does not allow us to deduce causality. As for the self-reported type of data collected, this could be prone to social desirability. Last but not least, this study was confined to a group of CPs in Lebanon, and therefore future studies should involve other healthcare professions to verify the psychometric credentials of the CBI-A. Lastly, future studies are needed to investigate other sources of evidence to further support its validity including relationships of CBI-A scores with other relevant consequences such as employability, intention to leave, physical illness, and medical errors….

## Strengths of the study

Within the current study, the psychometrical evaluation of the newly adapted CBI was tested on a sample of participants involved in direct contact with patients which represented a population of CPs at especially high risk of suffering from burnout-related difficulties. Notwithstanding the above limitations, our study findings support the use of CBI-A—a free-of-charge burnout inventory—to assess burnout among Lebanese CPs. It is also worth mentioning that, despite the many versions of the CBI in eight different languages and being tested over more than 15 different occupational groups, this is the first study that presents the Arabic version of the instrument and applied it to a sample of community pharmacists. Lastly, retaining all items of CBI will allow the comparison of burnout scores across different cultures or contexts [52].

## The implication of the study

Despite the challenges discussed above, the CBI-A is best for the assessment of exhaustion and does not confuse the experiences of burnout with other components, such as coping strategies or consequences of the syndrome. Due to this reason, the CBI facilitates identification and clarification of causal relationships of burnout, provides a better understanding of burnout, and helps to plan interventions to minimize unwanted consequences of burnout at the personal or professional level.

This study also contributes to the existing empirical evidence on the CBI-A psychometric properties as well as provides evidence on the differentiation of the three attributes of CBI. It also supported the CBI authors’ insights about the ability to use any of these subscales independently at any time to assess burnout. This is especially important for diverse groups like pharmacists where burnout may be experienced differently by different pharmacist profiles (CPs and clinical pharmacists) as the concentration for some practice settings might be work-related burnout rather than patient-related burnout or vice-versa. Lastly, this study would enable researchers and statisticians to conduct further studies in other healthcare settings to ensure objective assessment of burnout.

## Conclusion

Our study showed a high internal consistency, significant factor loadings for each subscale, and a low floor and ceiling effect. The adapted three-factor model of CBI-A showed also good construct validity and exhibited an acceptable fit to the data. As expected, CBI-A dimensions were found to be positively associated with extensive working hours and sleep disturbance while job satisfaction was negatively associated with CBI-A aspects. Mental health outcomes such as depression and anxiety were also found positively associated with CBI-A domains. Hence, the Arabic version of CBI is a psychometrically reliable and valid instrument for assessing burnout among CPs and thus could be a useful tool for assessing burnout syndrome among other healthcare workers.

## Supplementary Information

**Additional file 1: Appendix S1.** Socio-demographics characteristics of surveyed Lebanese community pharmacists (*N* = 387). **Appendix S2.** Clarity, comprehension, and face validity total, per question and subscale (%).

## Data Availability

After publication, the survey data will be made available based on reasonable requests to the corresponding author. A proposal with a detailed description of study objectives and a statistical analysis plan will be needed for the assessment of requests. Additional materials might also be required during the process of assessment.

## Abbreviations

CBI: Copenhagen Burnout Inventory
CBI-A: Arabic version of the Copenhagen Burnout Inventory
PP: Personal burnout
WB: Work-related burnout
CB: Client burnout
CPs: Community pharmacists
MBI: Maslach Burnout Inventory
COVID-19: Coronavirus disease-2019
SPSS: Statistical Package for Social Sciences
CFA: Confirmatory factor analysis
EFA: Exploratory factor analysis
HCWs: Health care workers
KMO: Kaiser–Meyer–Olkin
US: United States
CVI: Content Validity Index
CVR: Content validity
I-CVI: Item Validity Index
S-CVI: Content Validity Index at the scale level
HADS: Hospital Anxiety and Depression Scale
RMSEA: Root mean square error of approximation
SRMR: Standardized root mean square residual
GFI: Goodness-of-Fit Index
CFI: Comparative Fit Index
TLI: Tucker–Lewis Index
NFI: Normed Fit Index
Chisq/*df*: Chi-squared value/degree of freedom
