# Supplementary material for: Feeling the burn in the era of COVID-19: cross-cultural adaptation and validation of the Arabic version of the Copenhagen Burnout Inventory among community pharmacists
Source: J Pharm Policy Pract. 2022 Mar 17;15:21. doi: 10.1186/s40545-022-00419-x (PMC8929240; doi:10.1186/s40545-022-00419-x)
Supplement: Supplementary file 1 — Additional file 1: Appendix S1. Socio-demographics characteristics of surveyed Lebanese community pharmacists (N = 387). Appendix S2. Clarity, comprehension, and face validity total, per question and subscale (%). [file 40545_2022_419_MOESM1_ESM.docx]

# Feeling the burn in the era of COVID-19: Cross-cultural adaptation and validation of the Arabic version of the Copenhagen Burnout inventory among community pharmacists

## Additional materials:

| **Appendix S1: Socio-demographics characteristics of surveyed Lebanese community pharmacists (N=387)** | | |
| --- | --- | --- |
|  | **n** | **%** |
| **Gender** |  |  |
| Male | 179 | 46.30% |
| Female | 208 | 53.70% |
| **Age (years)** |  |  |
| Less than 40 y | 254 | 65.60% |
| ≥ 40 y | 133 | 34.40% |
| **Marital status** |  |  |
| Single and other* | 153 | 39.50% |
| Married/Engaged | 234 | 60.50% |
| **Pharmacy location** |  |  |
| North & Akkar | 48 | 12.40% |
| Mount Lebanon | 145 | 37.50% |
| Beirut | 60 | 15.50% |
| South & Nabatyeh | 79 | 20.40% |
| Bekaa & Baalbeck-Hermel | 55 | 14.20% |
| **Urbanicity (Residency)** |  |  |
| Rural | 132 | 34.10% |
| Urban | 255 | 65.90% |
| **Years of experience** |  |  |
| 0-10 years | 220 | 56.90% |
| More than 10 years | 167 | 43.20% |
| **Profile** |  |  |
| Staff pharmacist | 135 | 34.90% |
| Owner | 214 | 55.30% |
| Manager | 38 | 9.80% |
| **Highest education level** |  |  |
| BS pharmacy | 216 | 55.80% |
| Other (Master, PharmD, PhD...) | 171 | 44.20% |
| **Number of hours per week pharmacy is open** | | |
| Less than 50 hours | 40 | 10.40% |
| 50-120 hours | 316 | 81.60% |
| 7 days 24/24h | 31 | 8.00% |
| **Pharmacist working hours** |  |  |
| Less than 40 h |  |  |
| 40 hours or more | 155 | 40.10% |
| **Household income** | 232 | 59.90% |
| <2 Millions | 178 | 46.10% |
| >2 Millions | 209 | 53.9% |
| **Health status** |  |  |
| Fair and Below | 87 | 22.50% |
| Good and above | 300 | 77.50% |
| **Presence of dependent child** |  |  |
| No | 170 | 44.50% |
| Yes | 972 | 55.50% |
| **Presence of elderly people at home** |  |  |
| No | 188 | 48.60% |
| Yes | 199 | 51.40% |
| **Living with family member with comorbidities** |  |  |
| No | 157 | 40.60% |
| Yes | 230 | 59.40% |
| **Ever tested for COVID-19** |  |  |
| No | 90 | 23.30% |
| Yes | 297 | 76.70% |
| **Personal history of COVID-19 diagnosis** |  |  |
| No | 298 | 77.00% |
| Yes | 89 | 23.00% |
| **Family member/friend or colleague ever diagnosed with COVID-19** |  |  |
| No | 256 | 66.10% |
| Yes | 131 | 33.90% |
| **Colleague ever diagnosed with COVID-19** | | |
| No | 35 | 9.00% |
| Yes | 352 | 91.00% |
| *Note: n: Frequency, % Percentage,* **Other included divorced or widowed* | | |

| **Appendix S2: Clarity, comprehension, and face validity total, per question and subscale (%)** | | | | |
| --- | --- | --- | --- | --- |
|  |  | **Clarity**  **(%)** | **Comprehension**  **(%)** | **Face Validity index(%)** |
| **P** | **Personal Burnout** | **87.2** | **93.34** | **90.25** |
| P1 | How often do you feel tired? | 90 | 95 | 92.5 |
| P2 | How often are you physically exhausted? | 93 | 95 | 94 |
| P3 | How often are you emotionally exhausted? | 85 | 95 | 90 |
| P4 | How often do you think: ”I can’t take it anymore”? | 90 | 85 | 87.5 |
| P5 | How often do you feel worn out? | 80 | 95 | 87.5 |
| P6 | How often do you feel weak and susceptible to illness? | 85 | 95 | 90 |
| **W** | **Work-related burnout** | **85.71** | **95** | **90.35** |
| W1 | Is your work emotionally exhausting? | 90 | 95 | 92.5 |
| W2 | Do you feel burnt out because of your work? | 80 | 95 | 87.5 |
| W3 | Does your work frustrate you? | 85 | 95 | 90 |
| W4 | Do you feel worn out at the end of the working day? | 90 | 95 | 92.5 |
| W5 | Are you exhausted in the morning at the thought of another day at work? | 80 | 95 | 87.5 |
| W6 | Do you feel that every working hour is tiring for you? | 85 | 95 | 90 |
| W7 | Do you have enough energy for family and friends during leisure time? | 90 | 95 | 92.5 |
| **C** | **Client- related burnout** | **85** | **85** | **85** |
| C1 | Do you find it hard to work with clients? | 80 | 80 | 80 |
| C2 | Do you find it frustrating to work with clients? | 80 | 85 | 82.5 |
| C3 | Does it drain your energy to work with clients? | 85 | 90 | 87.5 |
| C4 | Do you feel that you give more than you get back when you work with clients? | 90 | 80 | 85 |
| C5 | Are you tired of working with clients? | 80 | 85 | 82.5 |
| C6 | Do you sometimes wonder how long you will be able to continue working with clients? | 95 | 90 | 92.5 |
|  | Overall CBI | 85.96 | 91.11 | 88.53 |
| P: Personal (6 items), W: Work (7 items), C: Client (6 items) | | | | |
